# A *Drosophila* model of cigarette smoke induced COPD identifies Nrf2 signaling as an expedient target for intervention

**DOI:** 10.18632/aging.101536

**Published:** 2018-08-27

**Authors:** Ruben Prange, Marcus Thiedmann, Anita Bhandari, Neha Mishra, Anupam Sinha, Robert Häsler, Philipp Rosenstiel, Karin Uliczka, Christina Wagner, Ali Önder Yildirim, Christine Fink, Thomas Roeder

**Affiliations:** 1 Kiel University, Zoology, Department of Molecular Physiology, Kiel, Germany; 2 Kiel University, IKMB, Kiel, Germany; 3 Research Center Borstel, Invertebrate Models, Borstel, Germany; 4 Comprehensive Pneumology Center, Institute of Lung Biology and Disease, Helmholtz Center Munich, German Research Center for Environmental Health, Munich, Germany; 5 University zu Lübeck, Institute for Cardiogenetics, Lübeck, Germany; 6 CPC-M, Member of the German Center for Lung Research (DZL); 7 Airway Research Center North (ARCN), Member of the German Center for Lung Research (DZL); * Equal contribution

**Keywords:** aging, life span, cigarette smoke, COPD, oltipraz

## Abstract

Chronic obstructive pulmonary disease (COPD) is among the most important causes of death. Signaling systems that are relevant for tissue repair and detoxification of reactive oxygen species or xenobiotics are thought to be impaired in lungs of patients suffering from this disease. Here, we developed a simple cigarette smoke induced *Drosophila* model of COPD based on chronic cigarette smoke exposure that recapitulates major pathological hallmarks of the disease and thus can be used to investigate new therapeutic strategies. Chronic cigarette smoke exposure led to premature death of the animals and induced a set of phenotypes reminiscent of those seen in COPD patients, including reduced physical activity, reduced body fat, increased metabolic rate and a substantial reduction of the respiratory surface. A detailed transcriptomic analysis revealed that especially the TGF-β, Nrf2 and the JAK/STAT signaling pathways are altered by chronic cigarette smoke exposure. Based on these results, we focused on Nrf2 signaling. A pharmacological intervention study performed with oltipraz, an activator of Nrf2 signaling, increased survival of cigarette smoke exposed animals significantly. Thus, the *Drosophila* COPD model recapitulates many major hallmarks of COPD and it is highly useful to evaluate the potential of alternative therapeutic strategies.

## Introduction

Chronic obstructive pulmonary disease (COPD) is the third leading cause of death by now. It is characterized by high morbidities and mortalities, both in high-income and low-income countries, thus leading to substantial socioeconomic burdens. As smoking and aging are the two most important risk factors for various chronic lung diseases including COPD, it is anticipated that the prevalence of COPD will rise substantially with the smoking exposure and increase in life expectancies [1,2]. Clinically, COPD is characterized by progressive airflow limitations and the development of emphysema, which describes the loss of functional parenchymal lung tissue [3]. This loss of functional alveolar structures that is one major hallmark of COPD [4], seems to be caused by inappropriate repair mechanisms operative in the diseased lung [5] and the inability to cope with increased levels of reactive oxygen species (ROS) in the organ. Premature functional aging of the lung seems to be a major aspect associated with or underlying COPD development [5,6]. Long-term cigarette smoke (CS) exposure is by far the most important risk factor, but the confrontation to other air pollutants, also contributes to disease development. Despite the great body of work invested in recent years, the understanding of the molecular mechanisms underlying COPD development is unsatisfactory. This lack of understanding comes along with a lack of causative therapeutic strategies, which sets an urgent need for the development of novel, alternative therapeutic options [3,7].

Beside the study of patient material, tailored animal models are usually the most informative tools to enhance our understanding of the molecular frameworks underlying the development of human diseases in general. For COPD related research, a series of complex rodent models based on chronic long-term exposure to cigarette smoke served for this purpose [8–10]. Using these toolboxes, pathological alterations causally related to disease development have been identified. The apparent inability to control tissue regeneration in the lung and to cope with increased levels of ROS have been identified as major disease promoting alterations [11]. In this context, findings about modifications in signaling systems associated with tissue repair and ROS protection have been most important. Wnt signaling appears to take a central position in this context, as it is directly associated with tissue repair processes. Using tailored animal models, it has been demonstrated that COPD development comes along or depends on the switch from canonical towards non-canonical Wnt signaling, thus reducing the repair capacity of the lung [12]. Moreover, other signaling systems such as the JAK/STAT pathway that are also relevant for repair mechanisms, have been identified as potential targets for anti-COPD therapies [13]. IL5 and IL6, which are cytokines leading to activation of STAT transcription factors, have been identified as valuable targets for novel intervention strategies, because they have been associated with COPD development [14–16]. In addition to systems that are directly involved in tissue repair mechanisms, those that react towards high ROS and/or xenobiotic levels are also deeply involved in COPD pathogenesis. Among these signaling systems, the Nrf2 pathway takes a central position as it is strongly activated by CS exposure and its impairment has been unequivocally linked to COPD development in patients [17].

Despite the substantial efforts that have been made to develop suited animal models and to understand the molecular framework underlying COPD development, an urgent need for novel tailored models and, based on them, novel therapeutic strategies derived therefrom is obvious [11,18]. Very simple animal models may represent promising new candidates that complete the currently available toolbox for COPD research. Most important among these simple models for human diseases are tailored, transgenic *Drosophila* systems that have been highly informative for enhancing our understanding of numerous human diseases [19,20]. The usage of *Drosophila* to understand the molecular framework underlying human diseases has recently been expanded to chronic lung disease including asthma and lung cancer [21–24]. Here, we present a simple *Drosophila* model of COPD that depends on chronic low-level CS exposure. It recapitulates the most important pathological hallmarks of the disease, such as premature death, reduced physical activity, enhanced metabolic rates and a significant reduction of respiratory surfaces. Moreover, we could show that CS exposure regulates especially TGF-β, Nrf2 and JAK/STAT signaling in the airway epithelium and based on this, we demonstrated that pharmacological activation of Nrf2 signaling increases survival significantly in those animals chronically treated with CS.

## RESULTS

**Figure 1:**
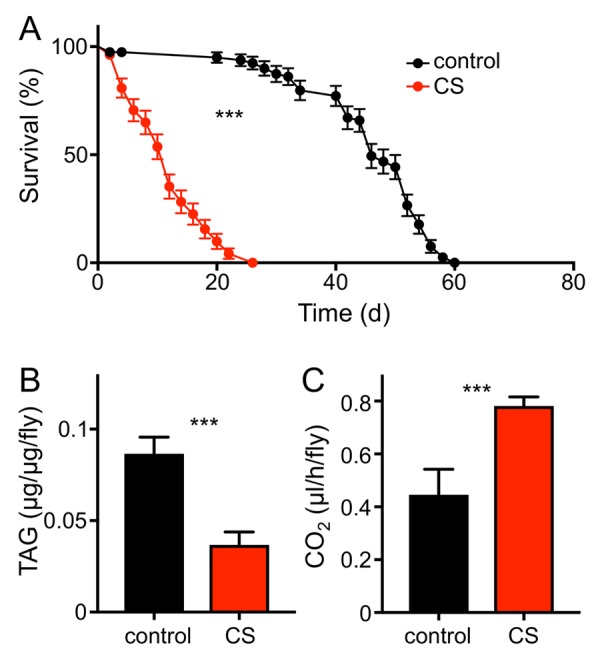
**CS exposure alters physiological parameters**. (**A**) Lifespan of chronically CS exposed *w^1118^* female flies (red symbols) compared to that of matching controls (black symbols). Statistical analysis revealed signficant differences (p< 0.0001, n =8). (**B**) Body fat contents of 10d CS exposed *w^1118^* females (red) and of matching controls (black). Mean values are significantly different (p<0.0001, n=5). (**C**) Mean metabolic rate of w^1118^ females after 10d of CS exposure and their matching controls (p<0.005, n = 11). Red symbols/bars mean CS exposed, black symbols/bars mean matching controls.

**Figure 2:**
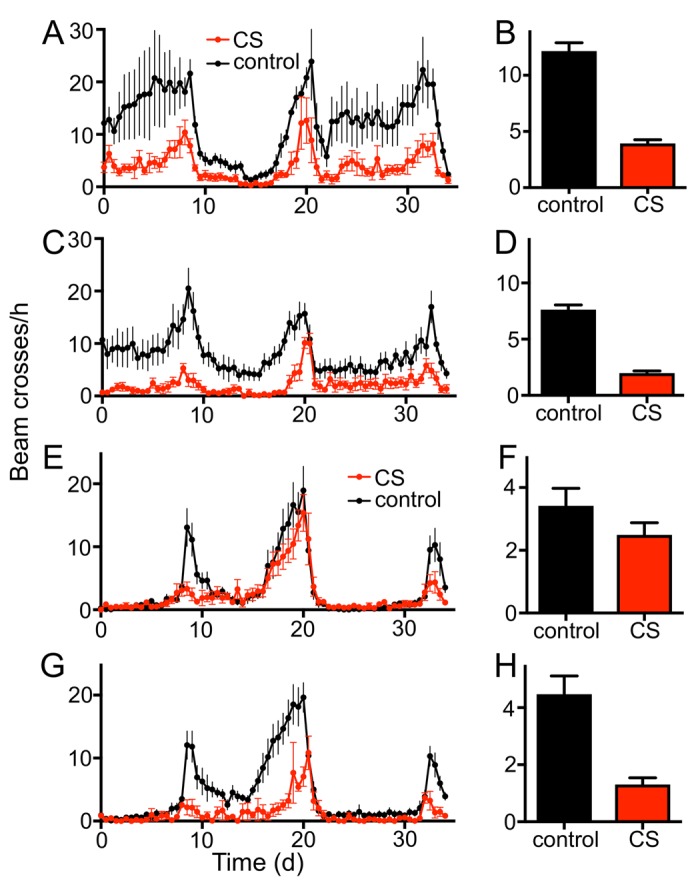
**Chronic CS exposure reduces the locomotor activity of male and female w^1118^ flies.** Effect of 7d CS exposure on the locomotor activity (**A**, 36 hour period, **B** mean values) of male flies (p<0.0001, n=16). 14d CS exposure affects movement activities substantially (**C**, 36 hour period, **D** mean values). Effects of 7d CS exposure on the locomotor activity (**E**, 36 hour period, **F** mean values) of female flies. The morning peak of 7d CS exposed female flies is almost completely diminished, while the overall activity is not significantly decreased. 14d of CS exposure affects the movement activities more severely (**G**, 36 hour period, H mean values, p<0.0001, n = 16). Red symbols/bars mean CS exposed, black symbols/bars mean matching controls.

To evaluate if the fruit fly *Drosophila melanogaster* can serve as a useful model for the analysis of CS induced morbidities, we confronted animals of different genotypes and developmental stages with cigarette smoke using different exposure regimens. Chronic short period exposure (app. 30 min per day) had a substantial effect on lifespan in wild type flies (female *Drosophila melanogaster*, *w^1118^*). This treatment reduced median lifespan statistically significantly by about 70% if compared with matching controls (Fig. 1A; p<0.001). In addition, we quantified the outcomes of CS exposure on different physiological and metabolic parameters. One major metabolic parameter is the body fat content. After 7 days of CS exposure, animals (females) had strongly reduced body fat levels if compared with untreated controls. This reduction was more than 50% of the control levels and it was statistically highly significant (Fig. 1B, p<0.001). We observed that the resting metabolic rates [25,26] of CS treated flies were almost twice as high as in non-treated matching controls (Fig. 1C, p<0.001). Moreover, animals confronted with daily doses of cigarette smoke showed a substantially reduced overall physical activity. Movement activities of CS treated and untreated animals were analyzed using the *Drosophila* activity monitoring system [27]. We observed sex-specific differences in the reaction towards CS exposure. Exposure to CS of only 1 week reduced the daily physical activity in males significantly (Fig. 2A, B) by about 60-70%. This reduction in movement activities was even more pronounced after 14d of CS exposure (Fig. 2C, D). In females, 7d exposure to CS led to a marginal decline in activity, which was not statistically significant (Fig. 2E, F). In contrast, 14d exposure induced a massive decline in physical activities also in females (Fig. 2G, H). A closer evaluation revealed that reduced activities are mainly observed in the major activity periods of flies, the so-called morning and evening peaks (Fig. 2A, C, E, G).

**Figure 3:**
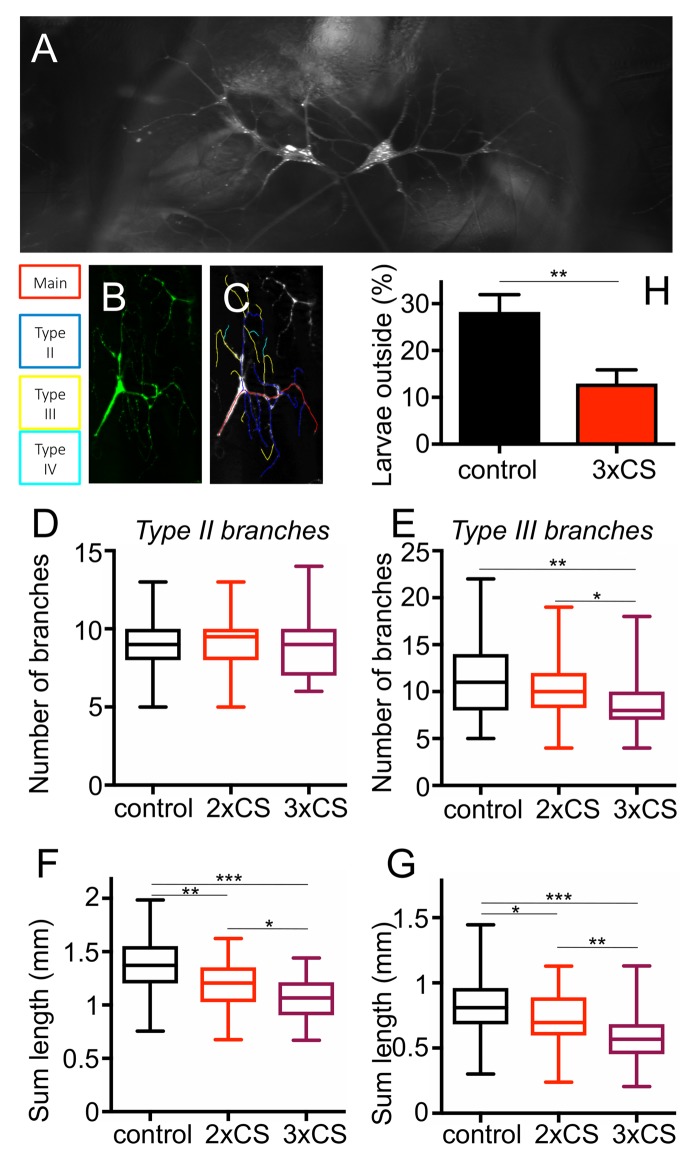
**Effect of CS exposure on terminal cells of 3^rd^ instar larvae.** Representative terminal cells of the dorsal branch in the third segment of *Drosophila* L3 larvae, where GFP was expressed under UAS control with a terminal cell specific GAL4 driver to visualize these tracheal terminal cells (**A**). For the analyses of the branching pattern, only one of the two terminal cells was analysed per replicate (**A**). Tracing of Type2, type3 and type4 branches was done with NeuronJ (**B**, **B’**). (**C**) The number of Type II branches was evaluated in control animals (black, those treated for 2 days with CS (red) and those treated for 3days with CS (violet). (**D**) as described for C, but focused on type III branches. Total lengths of type II (**E**) and type III (**F**) branches were measured. Also, the more frequent the smoke exposure, the shorter the length of Type II and III branches. To test for physiological changes after prolonged CS exposure, we analyzed the flight response of L3 larvae to reduced oxygen conditions. The percentage of larvae leaving the medium, after decreasing the concentration of O_2_ to 2.5-4%, was calculated. (* means p<0.05, ** means p<0.01, *** means, p<0.005).

To evaluate if cigarette smoke exposure induces structural changes in the airway system, we used *Drosophila* larvae as their airway system is highly amenable to a detailed structural analysis and modified the protocol for cigarette smoke exposure accordingly. Prolonged confrontation with cigarette smoke (2 or 3 days with 3 periods of 30 min daily) had a significant impact on the terminal structures of the airway system, which are those regions of the airway system devoted to gas exchange. These terminal cells have long gas filled protrusions to deliver oxygen to the different organs of the fly (Fig. 3A). In response to two different regimens of cigarette smoke exposure, the corresponding animals showed a reduced respiratory surface. To demonstrate this effect, we analyzed the overall numbers as well as the total lengths of type II and type III branches using an ImageJ plugin (Fig. 3B, C). For all experiments we observed similar trends, likewise whether we analyzed the parameters after 2 or 3 days, with more pronounced reductions after 3 days. The number of type II branches was not changed by this intervention (Fig. 3D), whereas the number of type III branches was significantly reduced after both, 2 and 3 days of CS exposure (Fig. 3E). After 3 days of CS exposure the reduction was almost 20%. Quantification of the total lengths of type II branches revealed a decrease from controls to animals treated for 2 days t. Moreover, a further decrease was observed if the animals treated for 3 days were included (Fig. 3F). Type III branches show an even greater reduction in total lengths after 2 and 3 days of treatment, which was up to 20%. All values were significantly different (Fig. 3G).

**Figure 4:**
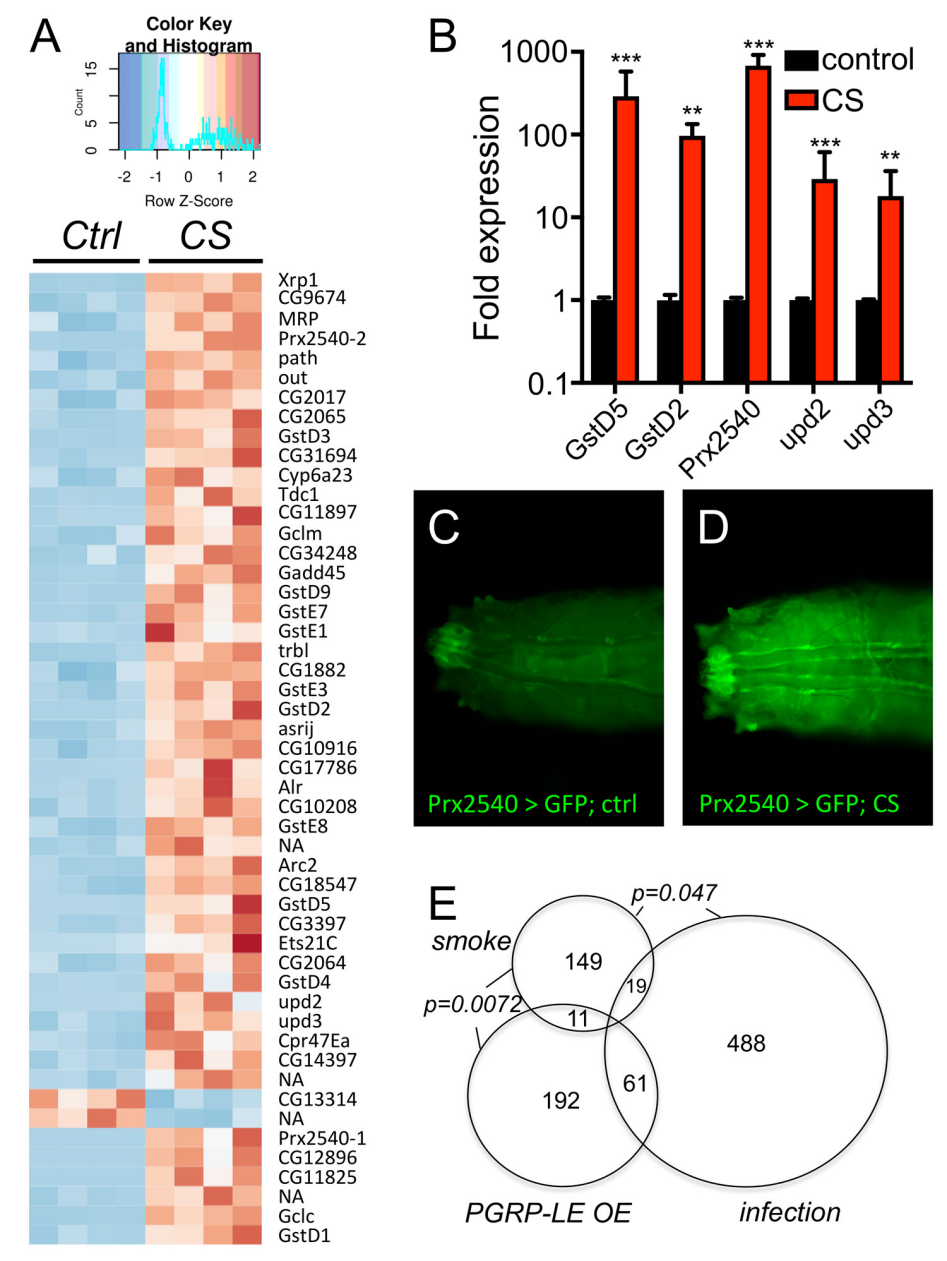
**Transcriptomic analyses of CS exposed trachea from 3^rd^ instar larvae.** (**A**) RNA sequencing experiment of trachea derived from nontreated (control) and CS exposed animals (CS) revealed a total of 143 genes significantly upregulated and 36 genes significantly downregulated in response to CS exposure (p<0.05). Shown is a heatmap with all 4 biological replicates comprising those 50 differentially transcribed genes with the most supportive p-values. qRT-PCR experiment to verify the differential transcription of selected genes (**B**). Transgenic animals carrying a Gal4 element under the control of the presumptive Prx2540 promoter (**C**, **D**) crossed to UAS-GFP flies and not exposed to CS (**C**) or exposed to CS (**D**). (**E**) Venn diagram analyses of those genes that were significantly regulated in respinse to CS exposure (smoke) compared with those regulated in response to infection (infection) and those regulated in response to ectopic PGRP-LE expression (PGRP-LE OE).

**Figure 5:**
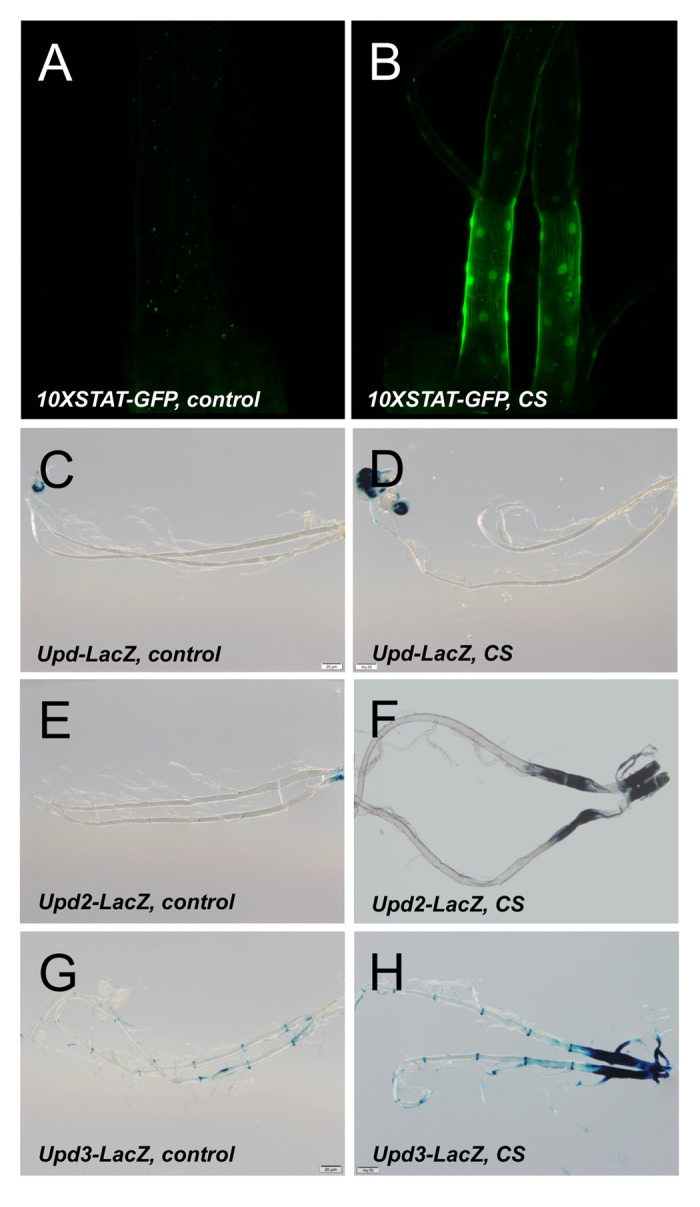
**Activation of the JAK/STAT signaling pathway by CS exposure*.*** A STAT reporter line (10XSTAT) tht was left untreated (**A**) or exposed to CS (**B**). LacZ staining of larvae carrying *upd*-Gal4, UAS-GFP (**C**, **D**), *upd2*-Gal4, UAS-GFP (**E**, **F**) or *upd3*-Gal4, UAS-GFP (**G**, **H**). Displayed are isoltaed trachea from non treated larvae (**C**, **E**, **G**) or those exposed to CS (**D**, **F**, **H**).

**Figure 6:**
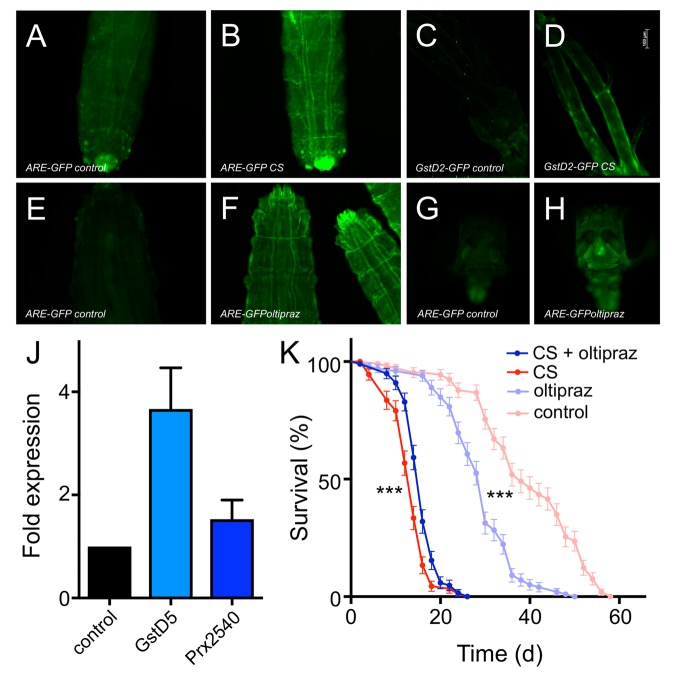
**Oltipraz ectopically activates Nrf2 signaling and increases lifespan of CS exposed flies.** CS exposure impacts the expression of GFP in Nrf2 reporter lines (A-D). 3^rd^ instar ARE-GFP larvae were left untreated (**A**) or exposed to CS (**B**). GstD2-GFP animals left untreated (**C**) or CS exposed (**D**). Impact of oltipraz on the activation of Nrf2 signaling in 3^rd^ instar larvae (E-H). 3^rd^ instar ARE-GFP larvae were left untreated (**E**) or exposed to oltipraz (**F**). GstD2-GFP animals left untreated (**G**) or treated with oltipraz (**H**). Effect of oltipraz on the transcript levels of canonical Nrf2 target genes (**J,** N=3, means ±S.D., n = 5, p > 0.01 for *gstd5*). Lifespan analysis of CS exposed animals (daily doses) that were left otherwise untreated (red symbols) or that were chronically confronted with oltipraz (blue symbols) (p<0.001, n = 5).

To evaluate the underlying molecular mechanisms, we performed a RNAseg analysis of untreated as well as CS exposed airways from 3^rd^ instar larvae of the same genotype. In total, 143 genes were statistically significantly upregulated and 36 genes were statistically significantly downregulated (Supplementry Table 1). Displayed is a heatmap of the top 50 differentially transcribed genes with regard to their statistical support (Fig. 4A, p<0.05). To further evaluate the validity of these RNAseq results, we selected a small group of genes showing increased transcript levels. The transcript level quantification was performed in independent experiments by qRT-PCR experiments. Both GST genes (*GstD2, GstD5*) that were analyzed showed several hundredfold transcript levels in response to CS exposure. A similar increase was observed for *Prx2540* and for the ligands of the JAK/STAT signaling pathway *upd2* and *upd3* (Fig. 4B). This observed increased expression was also seen in experiments using a promoter reporter line, where the presumptive *Prx2540* promoter drives expression of Gal4. Based on the Gal4/UAS system [28], the promoter activity could be monitored by utilizing GFP as the reporter. As expected, CS exposure induced expression of GFP that is mainly confined to the tracheal system (Fig. 4C, D). Given the complexity of the cohorts of significantly regulated genes, we identified common signatures using various approaches. We compared the cohorts of regulated genes identified in the current study with those sets of genes that were regulated in response to infection [29] or in response to ectopic activation of the IMD pathway [30] in the airways of larval *Drosophila* (Fig. 4E). Moreover, we searched for transcription factor binding sites in the presumptive promoter regions of these genes using Pscan [31] and performed a detailed gene ontology analysis using the DAVID program package [32,33]. Using Pscan, only 4 different transcription factor binding sites were significantly enriched in the cohorts of regulated genes. These are *Trl* (p=6X10^-7^), *Mad* (p=0.0052), *opa* (p=0.014) and *CTCF* (p=0.044). Moreover, we performed a GO analysis and retrieved significantly enriched KEGG pathways and GO terms. These are listed in SupplementaryTable 1 and comprise the enriched KEGG pathways: *glutathione metabolism* (p=1.1X10^-15^), *metabolism of xenobiotics by p450* (p=7.9X10^-16^), and *drug metabolism p450* (p=2.2X10^-14^). The GO term analyses (Supplementary Table S2) revealed some suspicious categories including *glutathione metabolic processes* (p=2.6X10*^-18^*), *response to oxidative stress* (p=0.0044), and *regulation of JAK-STAT cascade* (p=0.015) among others (Supplementary Table S1). Regulation of xenobiotic and glutathione metabolism is usually under the control of Nrf2 signaling. Thus, we focused on the two most suspicious signaling pathways, the Nrf2 and the JAK/STAT signaling systems for in-depth analyses. Thus, we employed reporter fly lines that specifically reflect activation of either pathway within the fly. We analyzed reporter lines that monitor activation of the JAK/STAT signaling pathway by subsequent production of GFP (1oXSTAT-GFP) and observed specific expression mostly confined to the trachea in CS exposed animals and here to those regions that are close to the spiracular openings (Fig. 5A, B). Moreover, we analyzed if the expression of the natural cytokines that activate the JAK/STAT signaling pathway are regulated. Here, no change could be observed for unpaired (*upd*, Fig. 5C, D), but strong activation in those parts of the tracheal system that are close to the spiracular openings could be observed for *upd2* (Fig. 5E, F) and *upd3* (Fig. 5G, H). We observed CS induced *upd3* expression also in distal parts of the tracheal system (Fig. 5E, F). In addition to the JAK/STAT system, we focused on the Nrf2 signaling system and used reporter lines that demonstrate a specific activation of this pathway (Fig. 6). In the so-called ARE-GFP line, CS exposure resulted in significant induction of GFP expression especially in the dorsal trunks (Fig. 6A, B), a very similar pattern was observed for the GstD2-Gfp line, which serves as a reliable reporter of Nrf2 activation (Fig. 6C, D). To manipulate this signaling system experimentally, we employed oltipraz, a specific Nrf2 activator, and treated larval and adult *Drosophila*. Oral application of this compound induced activation of the ARE-GFP reporter both in larvae (Fig. 6E, F) and adults (Fig. 6G, H). Quantitative evaluation of the transcript levels of two Nrf2 responsive genes, namely of *GstD2* and *Prx2540* revealed a specific increase in the tracheal tissue, implying for a specific activation of the corresponding signaling system (Fig. 6J). Taking this information into account, we evaluated if manipulation of this signaling pathway interferes with the organism’s sensitivity to CS exposure. To evaluate the effects of chronic oltipraz application independent of CS exposure, we treated control animals with this compound for their entire adult life and compared the effects with those animals that were only treated with the solvent. In this case, the application of oltipraz led to a significantly reduced life span (median survival reduced from 38d for untreated controls to 30d for oltipraz treated ones), implying that this oltipraz concentration had a slight but significant (p<0.0001) negative effect on life span in general (Fig. 6K). Moreover, we measured the life spans of cohorts of flies fed with oltipraz as well as of matching controls that were chronically exposed to CS. We find that administration of oltipraz prolonged median life spans in these CS treated animals from 14d to 16d, which is an increase of approximately 15% (Fig. 6K, p<0.0001).

## DISCUSSION

Chronic cigarette smoke exposure is the major cause of COPD. Although substantial steps forward have been made in the last years, our understanding of the molecular and cellular mechanisms that underlie disease progression is still not satisfactory [18]. This lack of understanding is mirrored by the lack of causal and successful therapeutic interventions [7]. To improve this unedifying situation, novel research strategies are mandatory. Thus, informative and druggable animal models are highly appreciated. The *Drosophila* model conceived here, shows a complex set of pathological phenotypes in response to chronic, low level CS exposure that resemble those seen in human COPD patients. Flies chronically exposed to CS have a strongly reduced life span, they show reduced physical activities, increased metabolic rates and a decreased body fat content. The occurrence of this set of phenotypes implies that CS exposure induces very similar alterations in flies and in human patients suffering from the disease [34]. Surprisingly, even the respiratory surfaces of CS exposed flies were significantly reduced, which is one major hallmark of COPD [4,35]. In patients, the gradual decline of respiratory net surface in the alveolar space finally leading to the development of emphysema, is the major pathological development in the disease [3,18,36].

Based on this surprising number of phenotypical similarities that encompass all major hallmarks of the disease, it is reasonable to utilize this seemingly unsuited model for further studies. Based on the transcriptomic studies performed with airway epithelia of CS exposed and non-treated flies, we observed very robust regulation of a restricted cohort of genes that comprise various genes that are deeply involved in responses towards xenobiotics and ROS. The abundant presence of both, ROS and xenobiotics, in the lungs of smokers are thought to be intimately connected to disease development [36,37]. Very impressive was the regulation of sets of genes in the airway system of *Drosophila* that are directly associated with the glutathione biology. The regulation and performance of the complex glutathione metabolism has been shown to be highly relevant in smoke-induced lung diseases. Glutathione and especially its biologically active form, reduced glutathione, is depleted upon CS exposure [38,39]. Presumably to counteract this depletion of biologically active glutathione metabolites, the expression of numerous genes coding for enzymes involved in glutathione metabolism is upregulated substantially in response to CS exposure. Moreover, failures to produce sufficient amounts of glutathione cause worsening of the inflammatory state of the lung, and numerous associations of genes of the glutathione metabolism have been linked with CS induced lung diseases or cancer [40,41].

Thus, this highly impressive increase in expression of numerous genes associated with the glutathione metabolism is in line with observations that have been made in patient material or in mammalian models of chronic CS exposure [42,43]. In addition, the regulation of genes, whose products are tightly associated with the detoxification of xenobiotics was very intriguing. Most important in this context were several cytochrome P450 enzymes (*Cyp*) that are also known to take a central position as detoxifying enzymes in the human lung and that are also upregulated in response to CS exposure in human or murine systems [44,45]. Although these *Cyp* genes are induced in response to xenobiotics, their activity is not per se positive, as some irritants and carcinogenic compounds need to be modified by the activity of specific *Cyps* to unfold their pathogenic potential [46,47].

Usually, these sets of genes are under the transcriptional control of stress responsive signaling systems such as the Nrf2 pathway. We could show that Nrf2 signaling is indeed substantially upregulated in response to CS exposure in well-defined parts of the tracheal system of the fly. Impairments in the Nrf2 mediated anti oxidative response in the lung is thought to be associated with the development of COPD [48]. Thus, it is reasonable to assume that manipulation of Nrf2 signaling in the airway epithelium is a promising intervention strategy to interfere with CS exposure induced pathologies [49–51]. We used oltipraz, a specific activator of Nrf2 signaling [52] and applied it together with the food. In flies, the small body size enables the activation of Nrf2 signaling in the airways, presumably due to diffusion processes. Moreover, this pharmacological treatment partially rescued the early lethality induced by CS exposure and enhanced median lifespan by approximately 15%. Although seemingly a minor effect, translated to the human situation, it would be equivalent to an increase in life span from 70 to 84 years. Moreover, it contrasts the overall negative effect seen by oltipraz administration on the life span in otherwise untreated animals. Thus, based on these results, it can be assumed that directed activation of Nrf2 signaling in the airways is a promising strategy to prevent or delay CS induced morbidities. Attempts to follow this approach in clinical trials failed so far [53]. Sulforaphane, which was used as a Nrf2 activator was administered orally in this clinical study. Orally applied Nrf2 activators not necessarily induce significant activation of this pathway in the lung. Our result also implied that oral administration of a general Nrf2 activation throughout the entire organism has severe negative effects. Based on our results, a revitalization of the concept to use Nrf2 activators as COPD therapeutics seems to be reasonable, but local application forms, such as inhalation therapies might be much more suited to achieve a protective effect.

Two other signaling systems were identified in this study that have unequivocally been linked to COPD development. These are the JAK/STAT- and the TGF-β-pathways. JAK/STAT signaling is centrally involved in repair processes in various tissues and thus represents a key signaling pathway that is tightly connected with the genesis of COPD [13]. Deregulated activity of this pathway or of upstream cytokine systems within the lung are highly relevant for disease development [13]. Both, cytokines such as IL5 or IL6, as well as the terminal modules of the signaling cascade, namely STAT1 and STAT3 have been associated with disease development. In the *Drosophila* system, we observed a very similar type of activation caused by CS exposure. Again, both, the transcription factors, but also the cytokines activating the system were upregulated in the airway epithelium. Interfering with this system at different levels has been shown to be a promising strategy for the treatment of COPD (IL5, IL6). The results of the current study imply that an autocrine positive feedback loop is operative under these experimental conditions and might thus contribute to disease development.

Among other functional categories, JAK/STAT signaling has been shown to be tightly associated with tissue regeneration processes. Tissue regeneration in general appears to be specifically impaired in the lungs of COPD patients, implying that manipulation of JAK/STAT signaling holds the potential to specifically interfere with these pathological developments. Upregulation of this highly important signaling pathway can have two different underlying mechanisms, the response to cellular damage caused by CS exposure in the treated airway epithelia or a specifically induced signaling system that is launched to prevent further cellular damage. Upregulation of this signaling system usually is a double-edged sword, it can be highly effective in safeguarding the cell’s survival even under stressful conditions, but a chronically deregulated pathway may lead to massive inflammation and finally to the development of cancer.

The *Drosophila* COPD model presented here is a valuable extension of the animal model toolbox that is currently in use to study this disease. Animal models of COPD are mainly based on CS exposed mice or other rodents [11,54,55]. Although their use has been highly informative, they have some drawbacks where alternative models can yield complementary information. Rodent COPD models can nicely reproduce major features of COPD, but long-term studies, especially those that comprise life span as a major read-out are almost impossible to perform. Moreover, these systems are not suited for high-throughput approaches aiming to evaluate the effectiveness of novel therapeutic interventions. Here, the fly model can step in as it allows quantifying the effects on life span in high-throughput settings, which can speed up the search for new therapeutic interventions substantially.

## MATERIALS AND METHODS

### Fly strains and fly husbandry

For most experiments, the *w^1118^* strain was used. Dsrf-Gal4 (gift of Jordi Casanova, Barcelona, Spain) has been described earlier [56], the UAS-6xGFP was made available from the Bloomington Stock Center (#52262). To isolate the presumptive Prx2540-promoter, a 1260 bp fragment upstream of the transcription start was amplified. This fragment was cloned into the pBPGAL4.2::VP16Uw vector (Addgene # 26228, Cambridge, USA) and injection into *w^1118^* was done by BestGene (Chino Hills, USA). Flies were cultured essentially as described earlier [26,57].

### Cigarette smoke exposure

All cigarette smoking exposure experiments were carried out in a smoking chamber, attached to a diaphragm pump. Common research 3R4F cigarettes (CTRP, Kentucky University, Lexington, USA) were used for all experiments. The vials containing flies or larvae, were capped with a monitoring grid to allow the cigarette smoke to diffuse into the vial. For experiments based on *Drosophila* larvae, 2^nd^ instar larvae were exposed to smoke three times a day for 30 min each, on two consecutive days. Adult flies (5-7days) were exposed to cigarette smoke once a day for 30 min on 5 days a week. Subsequent experiments with larvae were performed 30 min after the last cigarette exposure. Adult experiments were carried out 2 h after the last exposure.

### RNA isolation and RNA sequencing

For the gene expression analysis of 3^rd^ instar larvae trachea, larvae were dissected in cold PBS and transferred to RNA Magic (BioBudget, Krefeld, Germany) and processed essentially as described earlier [23] with slight modifications. The tissue was homogenized in a Bead Ruptor 24 (BioLab products, Bebensee, Germany) and the RNA was extracted by using the PureLink RNA Mini Kit (Thermo Fisher, Waltham, MA, USA) for phase separation with the RNA Magic reagent. An additional DNase treatment was performed following the on-column PureLink DNase treatment protocol (Thermo Fisher, Waltham, MA, USA).

The RNAseq reads were trimmed for low quality bases and adapters using the fastq illumina filter (http://cancan.cshl.edu/labmembers/gordon/fastq_illumina_filter/) and cutadapt (version 1.8.1) [58], respectively. Reads that were shorter than 50bp after the trimming were filtered out. The trimmed and filtered reads were mapped to the *D. melanogaster* reference genome (Release 6) [59] using TopHat (version 2.0.14) [60]. The expression counts of the genes were estimated using htseq [61] and the differential expression analysis was done using the Bioconductor package DESeq2 [62]. The transcription factor binding site enrichment and the Gene Ontology enrichment analyses of the differentially expressed genes were carried out using Pscan [31] and DAVID [32], respectively.

### Determination of body fat content and metabolic rates

To determine the body fat content of CS exposed *w^1118^* flies, whole female flies were essentially treated as described earlier [63]. In brief, flies were homogenized with a Bead Ruptor 24 (BioLabProducts, Bebensee, Germany). The lysate was heat inactivated for 5 min at 70°C and centrifuged for 3 min at 3000x g. The Sample was centrifuged again for 3 min at 2500xg and 50 µl of supernatant were transferred to 96-well plates. Absorbance was measured at 540 nm, 200 µl prewarmed Infinity Triglyceride solution (Fisher Scientific, Waltham, MA, USA) was added and incubated at 37 °C for 30 min. Absorbance was measured at 540 nm to determine the colorimetric reaction ratio.

To quantify the basic metabolic rate of smoked flies the amount of consumed CO_2_ was measured as readout for O_2_ usage. Therefore 5–7 d old mated female *w^1118^* flies were smoked for 10 d. The experimental procedure was performed as previously described [25,64]. Groups of 3 flies were analyzed for 2h periods. Data presented were calculated based on the volume of produced CO_2_ during the experimental phase. Data were obtained from at least 11 independent biological replicates.

### Lifespan measurements

Mated female *w^1118^* flies were collected and separated from males in the age of 5 to 7 days after hatching. 10 flies per vial were exposed to cigarette smoke for 30 min each day on 5 day per week. Prior smoke exposure, flies were transferred to empty vials to avoid contamination of the food. Flies were transferred to fresh vials every other day and the number of dead flies was counted accordingly. For the oltipraz treatment, flies were kept on media supplied with a final concentration of 400 µM oltipraz.

### Fluorescence microscopy

To visualize the activation of reporter lines, the trachea of 3^rd^ instar larvae were dissected in cold PBS and mounted in Roti-Mount (Roth, Karlsruhe, Germany) to stain the DNA. Images were acquired using a Zeiss Axio Imager Z1, equipped with an Apotome and an AxioCam MRm camera system (Zeiss, Oberkochen, Germany).

### Locomotor activity

For studying the activity of smoke exposed flies, a *Drosophila* Activity Monitoring (DAM) system was used [27]. 5-7 d old mated female *w^1118^* flies were smoked for 7 d and 14 d, respectively. Subsequently, flies were put into vials containing media made of 1.5% agar-agar and 5% sucrose. Activity was monitored for 2 d. Monitor files were processed with the DAM File scan software (TriKinetics, Waltham, USA) and the activity counts were binned to 30 min (n=8 with 10 flies each).

### Branching analysis

For comparison of the terminal cell branching pattern between CS exposed and control larvae, the terminal cell driver *dsrf*-Gal4 was crossed to the responder 20x UAS-6x *gfp*. Larvae were kept on 25 °C for five days until they reached L3 state. The treatment with cigarette smoke was conducted on day 4 and 5 or on day 3, 4 and 5 after mating for 2x3 or 3x3 CS exposure respectively. Each day 3 cigarettes were applied for 30 minutes with intervals of 3 hours. After the last treatment, larvae were used for the branching analysis. The protocol used for the branching pattern is based on the publication of Jones and Metzstein [65]. We analyzed the terminal cell of the dorsal branch in the 3^rd^ segment. The terminal cells were evaluated with the 10x objective. Five Z-Stacks of each larvae were taken to follow the branches into the periphery. Before using NeuronJ, the contrast of each image was increased by 2.5%. At least 28 independent measurements were used for analysis.

### Hypoxia sensitivity

For the Hypoxia sensitivity test *w^1118^* larvae were raised and treated as described for the branching experiment. On day five, 30 min after the last treatment, larvae were washed with 1x PBS and 10 larvae per treatment were put into a new vial. After 30 minutes the vials were put in a desiccator with a fitted O_2_ electrode. The oxygen was flushed out with nitrogen gas to a level of 2.5% - 4% O_2_. The flight response was monitored every 5 min for 20 minutes in total. For each time point the percentage of larvae that left the media was calculated. 17 independent replicates with 10 L3 larvae each were analysed.

### qRT-PCR

*w^1118^* larvae were raised on 25 °C for 5 days. The CS was applied on day 4 and 5 with 3 cigarettes per day. CS exposure lasted 30min. 30 min after the last cigarette treatment the trachea of the larvae were isolated in 1x PBS. The dissected tissue was transferred into RNA Magic (BioBudget, Krefeld, Germany). The isolation procedure is the same as for the RNAseq experiments. Isolated RNA was transcribed into cDNA with Superscript IV (Thermo Fisher Scientific, Carlsbad, USA). The cDNA was adjusted to 20 ng per well with 2x qPCRBio Sygreen Mix Hi Rox (PCRBiosystems, London, UK) and amplified for 40 cycles. Data was analyzed by ^∆∆^Ct and samples were normalized against rpl32. At least five independent samples were used.

### LacZ-staining

Trachea from 3^rd^ instar larvae were dissected in PBS and fixed for 25 min in 0,75% glutaraldehyde at RT. The tissue was washed twice for 5 min with PBS and pre-warmed (37°C ) staining solution (150 mM NaCl, 1 mM MgCl_2_, 3.1 mM K_4_[Fe(CN)_6_)], 3.1 mM K_3_[Fe(CN)_6_], 0.3% Triton X-100) supplied with 600 µM X-Gal was added. Trachea were incubated at 37 °C for 1h and imaged immediately after incubation.

## Supplementary Material

Supplementary Table 1

Supplementary Table 2
