# Supplementary material for: A Drosophila model of cigarette smoke induced COPD identifies Nrf2 signaling as an expedient target for intervention
Source: Aging (Albany NY). 2018 Aug 27;10(8):2122–35. doi: 10.18632/aging.101536 (PMC6128429; doi:10.18632/aging.101536)
Supplement: Supplementary Table 1 [file aging-10-101536-s001.docx]

**Supplementary Table 1. All genes with differential transcription (p<0.05) between control trachea and those isolated from animals exposed to cigarette smoke.**

| **gene_id** | **symbol** | **log2FoldChange** | **pvalue** | **padj** |
| --- | --- | --- | --- | --- |
| FBgn0033518 | Prx2540-2 | 6,655257748 | 5,36E-208 | 4,84E-204 |
| FBgn0033519 | CG11825 | 6,082121708 | 1,73E-131 | 5,21E-128 |
| FBgn0033521 | CG12896 | 5,6603073 | 5,82E-108 | 1,31E-104 |
| FBgn0033520 | Prx2540-1 | 5,491028095 | 7,42E-100 | 1,34E-96 |
| FBgn0010041 | GstD5 | 3,691596521 | 5,70E-44 | 6,43E-41 |
| FBgn0010039 | GstD3 | 3,309934172 | 2,08E-71 | 3,13E-68 |
| FBgn0010038 | GstD2 | 3,246308113 | 1,05E-36 | 9,52E-34 |
| FBgn0040319 | Gclc | 3,154365581 | 1,28E-132 | 5,79E-129 |
| FBgn0259977 | Tdc1 | 2,967359676 | 9,02E-42 | 9,04E-39 |
| FBgn0063493 | GstE7 | 2,758003639 | 1,84E-32 | 1,51E-29 |
| FBgn0010040 | GstD4 | 2,589383287 | 1,08E-20 | 4,87E-18 |
| FBgn0030904 | upd2 | 2,407356673 | 4,35E-17 | 1,64E-14 |
| FBgn0037975 | CG3397 | 2,224851424 | 2,09E-20 | 8,98E-18 |
| FBgn0033204 | CG2065 | 2,111550448 | 6,80E-30 | 4,72E-27 |
| FBgn0038020 | GstD9 | 2,073958721 | 6,54E-31 | 4,92E-28 |
| FBgn0033980 | NA | 2,059293904 | 2,05E-15 | 6,85E-13 |
| FBgn0063492 | GstE8 | 1,988789993 | 7,49E-17 | 2,70E-14 |
| FBgn0034335 | GstE1 | 1,95070664 | 2,05E-14 | 6,59E-12 |
| FBgn0053542 | upd3 | 1,93520144 | 2,02E-13 | 6,29E-11 |
| FBgn0033928 | Arc2 | 1,846143312 | 3,85E-26 | 2,32E-23 |
| FBgn0063497 | GstE3 | 1,822042205 | 3,67E-19 | 1,44E-16 |
| FBgn0032914 | CG14397 | 1,816302402 | 2,32E-10 | 5,15E-08 |
| FBgn0259834 | out | 1,808827414 | 8,93E-24 | 4,47E-21 |
| FBgn0033153 | Gadd45 | 1,800033411 | 2,88E-23 | 1,37E-20 |
| FBgn0267408 | NA | 1,755361601 | 5,73E-25 | 3,04E-22 |
| FBgn0033597 | Cpr47Ea | 1,73920416 | 3,54E-10 | 7,56E-08 |
| FBgn0005660 | Ets21C | 1,686775502 | 1,52E-09 | 2,91E-07 |
| FBgn0267635 | NA | 1,616973493 | 9,64E-09 | 1,67E-06 |
| FBgn0266705 | NA | 1,591241969 | 8,58E-09 | 1,55E-06 |
| FBgn0036007 | path | 1,575012432 | 7,65E-16 | 2,65E-13 |
| FBgn0063494 | GstE6 | 1,482395251 | 2,03E-08 | 3,40E-06 |
| FBgn0037973 | CG18547 | 1,463930253 | 3,23E-28 | 2,08E-25 |
| FBgn0040609 | CG3348 | 1,46224182 | 1,37E-07 | 1,99E-05 |
| FBgn0037850 | CG14695 | 1,434086469 | 2,56E-07 | 3,55E-05 |
| FBgn0085277 | CG34248 | 1,38832712 | 6,47E-10 | 1,30E-07 |
| FBgn0039644 | CG11897 | 1,387464349 | 1,05E-12 | 2,86E-10 |
| FBgn0033978 | Cyp6a23 | 1,369726203 | 3,60E-10 | 7,56E-08 |
| FBgn0037934 | CG6830 | 1,355795449 | 3,22E-08 | 5,19E-06 |
| FBgn0032456 | MRP | 1,302751559 | 4,04E-10 | 8,29E-08 |
| FBgn0063495 | GstE5 | 1,294768493 | 1,05E-06 | 0,000131371 |
| FBgn0031068 | Alr | 1,27861489 | 8,45E-13 | 2,38E-10 |
| FBgn0040262 | Ugt36Ba | 1,262929765 | 9,18E-08 | 1,39E-05 |
| FBgn0028978  www.aging-us.com 1 AGING | trbl | 1,240511738 | 3,86E-25 | 2,18E-22 |
| FBgn0085310 | CG34281 | 1,235120999 | 1,50E-05 | 0,001411977 |
| FBgn0034394 | CG15096 | 1,233668574 | 2,97E-07 | 4,06E-05 |
| FBgn0046114 | Gclm | 1,225540658 | 3,63E-11 | 8,86E-09 |
| FBgn0033205 | CG2064 | 1,214330755 | 1,53E-10 | 3,55E-08 |
| FBgn0039118 | CG10208 | 1,206466801 | 3,89E-12 | 1,00E-09 |
| FBgn0010651 | l(2)08717 | 1,198877544 | 4,51E-08 | 7,13E-06 |
| FBgn0000473 | Cyp6a2 | 1,189591754 | 8,22E-06 | 0,000823734 |
| FBgn0042206 | GstD10 | 1,160899201 | 3,48E-05 | 0,003044632 |
| FBgn0261113 | Xrp1 | 1,151579425 | 3,77E-50 | 4,86E-47 |
| FBgn0037974 | CG12224 | 1,129145323 | 3,57E-06 | 0,00037468 |
| FBgn0033226 | CG1882 | 1,120832957 | 1,47E-11 | 3,68E-09 |
| FBgn0033926 | Arc1 | 1,098962745 | 0,000112232 | 0,008232154 |
| FBgn0010042 | GstD6 | 1,096834574 | 0,000130865 | 0,009230472 |
| FBgn0033732 | CG13157 | 1,073625189 | 4,89E-06 | 0,000501614 |
| FBgn0039167 | CG17786 | 1,061215282 | 6,65E-10 | 1,30E-07 |
| FBgn0001149 | GstD1 | 1,054446288 | 3,09E-12 | 8,21E-10 |
| FBgn0031689 | Cyp28d1 | 1,026263523 | 1,58E-05 | 0,001472327 |
| FBgn0028424 | JhI-26 | 1,023861651 | 0,000224694 | 0,014377228 |
| FBgn0037391 | CG2017 | 0,998803562 | 5,20E-13 | 1,51E-10 |
| FBgn0038315 | CG14866 | 0,994075517 | 0,000513743 | 0,028435491 |
| FBgn0259711 | CG42365 | 0,987890805 | 1,61E-05 | 0,001477865 |
| FBgn0035157 | CG13894 | 0,980342237 | 0,000254688 | 0,01606852 |
| FBgn0041607 | asparagine-synthetase | 0,976111682 | 0,000325691 | 0,01958924 |
| FBgn0039257 | tnc | 0,971543291 | 0,000111653 | 0,008232154 |
| FBgn0039316 | CG11893 | 0,968600916 | 0,0005059 | 0,028349241 |
| FBgn0051288 | CG31288 | 0,968079137 | 0,000687811 | 0,036289055 |
| FBgn0033599 | CG13223 | 0,953821462 | 0,000451275 | 0,026267088 |
| FBgn0037151 | CG7130 | 0,942170179 | 1,63E-05 | 0,001483202 |
| FBgn0266088 | NA | 0,939820576 | 7,70E-05 | 0,006146785 |
| FBgn0036663 | CG9674 | 0,936711828 | 2,96E-20 | 1,21E-17 |
| FBgn0034756 | Cyp6d2 | 0,93137034 | 0,000215842 | 0,014214054 |
| FBgn0035513 | Cpr64Ad | 0,904096939 | 0,000462679 | 0,026758279 |
| FBgn0038455 | CG14907 | 0,885405205 | 3,49E-06 | 0,000370566 |
| FBgn0038475 | Keap1 | 0,863795732 | 0,000350355 | 0,020795411 |
| FBgn0041184 | Socs36E | 0,855183551 | 1,32E-05 | 0,001253093 |
| FBgn0036588 | CG13068 | 0,843358233 | 0,000803152 | 0,041643893 |
| FBgn0033945 | CG12868 | 0,840254634 | 0,000979377 | 0,049362765 |
| FBgn0260747 | CG5010 | 0,83701902 | 1,59E-06 | 0,000188545 |
| FBgn0034312 | CG10916 | 0,833769468 | 2,89E-13 | 8,68E-11 |
| FBgn0030263 | CG2076 | 0,832852102 | 1,83E-05 | 0,001638468 |
| FBgn0034354 | GstE11 | 0,815128234 | 2,54E-08 | 4,17E-06 |
| FBgn0034709 | Swim | 0,809352113 | 5,92E-05 | 0,004853739 |
| FBgn0036502 | CG7841 | 0,805829475 | 5,04E-05 | 0,004252764 |
| FBgn0028543 | NimB2 | 0,795440521 | 1,04E-06 | 0,000131371 |
| FBgn0031643  www.aging-us.com 2 AGING | CG3008 | 0,792098332 | 1,83E-06 | 0,0002094 |
| FBgn0029866 | CG3842 | 0,789622293 | 3,56E-05 | 0,003087673 |
| FBgn0051694 | CG31694 | 0,781964008 | 2,34E-10 | 5,15E-08 |
| FBgn0085438 | CG34409 | 0,780838399 | 0,000943804 | 0,048107327 |
| FBgn0263316 | Mrp4 | 0,772466336 | 8,96E-06 | 0,000868895 |
| FBgn0262524 | ver | 0,747337817 | 0,000284204 | 0,017442766 |
| FBgn0039525 | CG5646 | 0,733111702 | 1,34E-06 | 0,000160814 |
| FBgn0027836 | Dgp-1 | 0,731067844 | 2,85E-06 | 0,000317788 |
| FBgn0046763 | CG17278 | 0,728510453 | 0,000432998 | 0,025366909 |
| FBgn0037354 | CG12171 | 0,724073576 | 0,000127333 | 0,009117456 |
| FBgn0036702 | CG6512 | 0,72151781 | 0,00021958 | 0,014355457 |
| FBgn0260755 | CG42553 | 0,719059634 | 1,26E-06 | 0,000156051 |
| FBgn0052549 | CG32549 | 0,717728318 | 0,000625828 | 0,033608429 |
| FBgn0013953 | Esp | 0,707600288 | 0,000122442 | 0,008837377 |
| FBgn0015351 | CG14906 | 0,661038434 | 0,000110092 | 0,008232154 |
| FBgn0034793 | asrij | 0,655498885 | 3,45E-09 | 6,36E-07 |
| FBgn0030189 | CG2909 | 0,646716264 | 3,72E-05 | 0,003199899 |
| FBgn0023407 | B4 | 0,629033531 | 1,68E-08 | 2,86E-06 |
| FBgn0020388 | Gcn5 | 0,623435388 | 0,000345978 | 0,020671608 |
| FBgn0034915 | eIF6 | 0,618527887 | 0,000100727 | 0,007636595 |
| FBgn0010611 | Hmgs | 0,609705973 | 0,000224407 | 0,014377228 |
| FBgn0002641 | mal | 0,602541562 | 0,000121246 | 0,008821629 |
| FBgn0020653 | Trxr-1 | 0,597841921 | 3,01E-06 | 0,000330953 |
| FBgn0031418 | CG3609 | 0,592909028 | 8,53E-06 | 0,000836264 |
| FBgn0040259 | Ugt86Da | 0,588212355 | 2,20E-07 | 3,14E-05 |
| FBgn0001220 | Hsc70-5 | 0,562334417 | 8,69E-05 | 0,006757273 |
| FBgn0260945 | Atg1 | 0,559549085 | 5,29E-06 | 0,000535977 |
| FBgn0031568 | CG10019 | 0,553176996 | 0,000110999 | 0,008232154 |
| FBgn0037852 | Tpc1 | 0,552140859 | 2,23E-07 | 3,14E-05 |
| FBgn0031307 | MFS3 | 0,541355991 | 0,000150553 | 0,01036863 |
| FBgn0010786 | l(3)02640 | 0,535713599 | 7,80E-05 | 0,006170164 |
| FBgn0031498 | CG17260 | 0,528943623 | 0,000481929 | 0,027345679 |
| FBgn0029664 | CG10802 | 0,521310304 | 9,59E-05 | 0,007334212 |
| FBgn0011774 | Irbp | 0,508706787 | 0,0007752 | 0,040426922 |
| FBgn0263782 | Hmgcr | 0,506356204 | 3,38E-06 | 0,000363237 |
| FBgn0032393 | CG12264 | 0,505130433 | 0,000578342 | 0,031432552 |
| FBgn0010803 | Aats-trp | 0,502169131 | 4,79E-05 | 0,004074312 |
| FBgn0026737 | CG6171 | 0,494063811 | 0,000231186 | 0,014688422 |
| FBgn0028540 | CG9008 | 0,493283153 | 8,95E-07 | 0,000115296 |
| FBgn0266268 | NA | 0,490952359 | 9,59E-08 | 1,42E-05 |
| FBgn0033761 | CG8778 | 0,486927589 | 6,41E-05 | 0,005206054 |
| FBgn0037071 | CG7632 | 0,484784671 | 0,000953101 | 0,048308287 |
| FBgn0036759 | CG5577 | 0,482289008 | 0,000186949 | 0,012681596 |
| FBgn0015623 | Cpr | 0,475288262 | 8,48E-06 | 0,000836264 |
| FBgn0034718 | wdp | 0,474815855 | 1,01E-05 | 0,000969183 |
| FBgn0015541 | sda | 0,474751936 | 0,000144544 | 0,010031365 |
| FBgn0035039  www.aging-us.com 3 AGING | CG3608 | 0,473415531 | 0,000576027 | 0,031432552 |
| FBgn0027079 | Aats-val | 0,461174455 | 0,000275249 | 0,017208925 |
| FBgn0051793 | CG31793 | 0,450121915 | 0,000562798 | 0,030960759 |
| FBgn0002891 | mus205 | 0,4272274 | 2,46E-06 | 0,000277334 |
| FBgn0033918 | CG8531 | 0,415761877 | 5,12E-05 | 0,004280445 |
| FBgn0034948 | Gadd34 | 0,363687367 | 0,000665287 | 0,035307195 |
| FBgn0037327 | PEK | 0,349863758 | 0,000299312 | 0,018245878 |
| FBgn0014141 | cher | 0,344389027 | 8,54E-05 | 0,006700604 |
| FBgn0002069 | Aats-asp | 0,331842804 | 0,000314912 | 0,019068008 |
| FBgn0036891 | CG9372 | 0,27671333 | 8,15E-07 | 0,000106608 |
| FBgn0250848 | 26-29-p | -0,217656134 | 0,000510101 | 0,028408202 |
| FBgn0262739 | AGO1 | -0,263862335 | 0,00091222 | 0,046761648 |
| FBgn0259176 | bun | -0,287447507 | 0,000712895 | 0,037393848 |
| FBgn0085450 | Snoo | -0,312796886 | 0,000281778 | 0,017412331 |
| FBgn0052428 | CG32428 | -0,342813294 | 0,000583166 | 0,031504952 |
| FBgn0032170 | CG4658 | -0,346640956 | 1,32E-06 | 0,000160814 |
| FBgn0031816 | CG16947 | -0,401340462 | 0,000477032 | 0,027239129 |
| FBgn0265623 | NA | -0,405836314 | 3,48E-05 | 0,003044632 |
| FBgn0026144 | CBP | -0,475447496 | 0,000130958 | 0,009230472 |
| FBgn0033631 | Sod3 | -0,491906476 | 0,000276579 | 0,017208925 |
| FBgn0038053 | CG18549 | -0,541999249 | 0,000139607 | 0,009763838 |
| FBgn0264834 | NA | -0,561544873 | 0,000213489 | 0,01416246 |
| FBgn0037409 | Osi24 | -0,562845734 | 5,76E-05 | 0,004771622 |
| FBgn0027790 | GV1 | -0,61559649 | 3,30E-07 | 4,38E-05 |
| FBgn0031515 | CG9664 | -0,658418469 | 0,00040076 | 0,023631714 |
| FBgn0267495 | NA | -0,677789832 | 4,78E-08 | 7,43E-06 |
| FBgn0032400 | CG6770 | -0,757880726 | 0,00046655 | 0,026810285 |
| FBgn0039864 | CG11550 | -0,792520118 | 7,34E-05 | 0,0059104 |
| FBgn0034808 | CG9896 | -0,809112613 | 1,75E-05 | 0,001580732 |
| FBgn0265575 | NA | -0,817016834 | 3,33E-06 | 0,000361927 |
| FBgn0035625 | Blimp-1 | -0,82223686 | 1,79E-06 | 0,000207591 |
| FBgn0052365 | CG32365 | -0,876648312 | 0,000641781 | 0,034261224 |
| FBgn0263973 | jv | -0,893143945 | 0,000196541 | 0,013232767 |
| FBgn0038398 | sxe2 | -0,897756238 | 4,04E-06 | 0,000419284 |
| FBgn0262112 | sro | -0,916404685 | 0,000494835 | 0,027902501 |
| FBgn0259101 | CG42249 | -0,930594936 | 0,000836532 | 0,043126801 |
| FBgn0037114 | Cpr78E | -0,989213571 | 0,000224471 | 0,014377228 |
| FBgn0016076 | vri | -0,989835826 | 1,67E-06 | 0,000195343 |
| FBgn0035949 | CG13314 | -1,02293734 | 1,63E-09 | 3,06E-07 |
| FBgn0263406 | NA | -1,038333487 | 0,000211019 | 0,014102314 |
| FBgn0052213 | CG32213 | -1,064634405 | 0,000152812 | 0,010444495 |
| FBgn0038005 | Cyp313a5 | -1,090404757 | 3,19E-07 | 4,30E-05 |
| FBgn0034499 | Cpr56F | -1,123778715 | 8,93E-05 | 0,006885303 |
| FBgn0263617 | NA | -1,143395873 | 5,23E-11 | 1,24E-08 |
| FBgn0025456 | CREG | -1,414024254 | 9,02E-09 | 1,60E-06 |
| FBgn0040733 | CG15068 | -1,531048078 | 9,24E-08 | 1,39E-05 |

www.aging-us.com 4 AGING
