# Supplementary material for: A Drosophila model of cigarette smoke induced COPD identifies Nrf2 signaling as an expedient target for intervention
Source: Aging (Albany NY). 2018 Aug 27;10(8):2122–35. doi: 10.18632/aging.101536 (PMC6128429; doi:10.18632/aging.101536)
Supplement: Supplementary Table 2 [file aging-10-101536-s002.docx]

**Supplementary Table 2. GO-term analysis of differentially transcribed genes and KEGG-pathway analysis.**

| **Biological process** | **p-value** | **FDR** | **Genes** |
| --- | --- | --- | --- |
| Glutathione metabolic process | 2.6 E ^-18^ | 8.2 E^-16^ | Gclc, GstD1, GstD10, GstD2, GstD3, GstD4, GstD5, GstD6, GstD9, GstE1, GstE11, GstE3, GstE5, GstE6, GstE7, GstE8 |
| Oxidation-reduction process | 3.0 E ^-7^ | 4.9 E^-4^ | AOX1, Alr, Cyp6a23, Cyp6a20, Cyp28d1, CG12896, Cyp313a5, CG18547, CG3397, CG3609, Cyp6d2, CG9674, CREG, Cpr, Cyp6a2, Hmgcr, Prx3540-1, Prx3540-2, Sod3, Trxr-1 |
| Transmembrane transport | 6.0 E ^-4^ | 6.2 E^-2^ | CG10019, CG11897, CG13223, CG31793, Esp, MFS14, MFS3, Mrp4, MRP, Tpc1, out |
| Response to oxidative stress | 4.5 E ^-3^ | 3.0 E^-1^ | Atg1, CG12896, Keap1, GstE1, Mrp4, Sod3 |
| Regulation of JAK-STAT cascade | 1.5 E ^-2^ | 6.1 E^-1^ | asrij, Socs36E, upd3 |
| Cell redox homeostasis | 2.3 E ^-2^ | 7.1 E^-1^ | CG12896, Prx2540-1, Prx2540-2, Trxr-1 |
| Positive regulation of JAK-STAT cascade | 2.9 E ^-2^ | 7.5 E^-1^ | CG31694, upd2, upd3 |
| Insecticide catabolic process | 2.9 E ^-2^ | 7.5 E^-1^ | Cyp28d1, Cyp6d2, Cyp6a2 |
| Cell differentiation | 3.5 E ^-2^ | 7.6 E^-1^ | Socs36E, sda |
| Anion transport | 4.2 E ^-2^ | 7.9 E^-1^ | CG15096, MFS14, MFS3 |
| Response to DDT | 4.2 E ^-2^ | 7.9 E^-1^ | Cyp28d1, Cyp6d2, Cyp6a2 |
| Intestinal epithelial structure maintenance | 4.6 E ^-2^ | 7.8 E^-1^ | upd2, upd3 |

| **Molecular function** | **p-value** | **FDR** | **Genes** |
| --- | --- | --- | --- |
| Glutathione peroxidase activity | 7.0 E^-23^ | 1.2 E^-20^ | GstD1, GstD10, GstD2, GstD3, GstD4, Gstd5, GstD6, GstD9, GstE1, GstE11, GstE3, GstE5, GstE6, GstE7, GstE8, Prx2540-1, Prx2540-2 |
| Glutathione transferase activity | 1.4 E^-16^ | 9.3 E^-15^ | GstD1, GstD10, GstD2, GstD3, GstD4, Gstd5, GstD6, GstD9, GstE1, GstE11, GstE3, GstE5, GstE6, GstE7, GstE8, |
| Oxidoreductase activity | 3.5 E^-7^ | 1.9 E^-5^ | Cyp6a23, Cyp6a20, Cyp28d1, CG12171, CG12224, CG18547, CG2064, CG2065, CG3397, CG3609, CG3842, Cyp6d2, CREG, Cyp6a2, sro |
| Peroxiredoxin activity | 1.8 E^-3^ | 7.3 E^-2^ | CG12896, Prx25040-1, Prx2540-2 |
| Iron ion binding | 7.0 E^-3^ | 1.3 E^-1^ | AOX1, Cyp6a23, Cyp6a20, Cyp28d1, Cyp313a5, Cyp6d2, CG9674, Cyp6a2 |
| Monooxygenase activity | 7.0 E^-3^ | 1.8 E^-1^ | Cyp6a23, Cyp6a20, Cyp28d1, Cyp313a5, Cyp6d2,Cyp6a2 |
| Oxidoreductase activity, acting on paired donors | 8.1 E^-2^ | 1.8 E^-1^ | Cyp6a23, Cyp6a20, Cyp28d1, Cyp313a5, Cyp6d2,Cyp6a2 |
| Flavin adenine dinucleotide binding | 9.0 E^-3^ | 1.7 E^-1^ | AOX1, Alr, CG9674, Cpr, Trxr-1 |
| FMN binding | 9.4 E^-3^ | 1.6 E^-1^ | CG9674, CREG, Cpr |
| Peroxidase activity | 1.5 E^-2^ | 2.3 E^-1^ | CG12896, Prx25040-1, Prx2540-2 |
| ATPase activity, coupled to transmembrane movement of substances | 2.1 E^-2^ | 2.7 E^-1^ | CG11897, CG31793, Mrp4, MRP |
| ATP binding | 2.7 E^-2^ | 3.1 E^-1^ | Aats-asp, Atg1, CG10802, CG11897, CG3008, CG31793, CG6512, CG9664, Gclc, Hsc70-5, Irbp, Mrp4, MRP, Aats-trp, Aats-val, Adck, PEK, trbl |
| Heme binding | 3.5 E^-2^ | 3.6 E^-1^ | Cyp6a23, Cyp6a20, Cyp28d1, Cyp313a5, Cyp6d2,Cyp6a2 |
| Glutamate-cysteine ligase activity  www.aging-us.com 1 AGING | 4.0 E^-2^ | 3.9 E^-1^ | Gclc, Gclm |

| **KEGG pathway** | **p-value** | **FDR** | **Genes** |
| --- | --- | --- | --- |
| Metabolism of xenobiotics by cytochrome P450 | 7.9 E^-16^ | 3.0 E^-14^ | Ugt36Ba, CG3609, GstD1, GstD10, GstD2, GstD3, GstD4, Gstd5, GstD6, GstD9, GstE1, GstE11, GstE3, GstE5, GstE6, GstE7, GstE8 |
| Glutathione metabolism | 1.1 E^-15^ | 1.9 E^-14^ | Gclc, Gclm, GstD1, GstD10, GstD2, GstD3, GstD4, Gstd5, GstD6, GstD9, GstE1, GstE11, GstE3, GstE5, GstE6, GstE7, GstE8 |
| Drug metabolism – cytochrome P450 | 2.2 E^-14^ | 2.8 E^-13^ | Ugt36Ba, GstD1, GstD10, GstD2, GstD3, GstD4, Gstd5, GstD6, GstD9, GstE1, GstE11, GstE3, GstE5, GstE6, GstE7, GstE8 |

Analysis was performed with the DAVID program package (Huang DW, Sherman BT, Lempicki RA. Systematic and integrative analysis of large gene lists using DAVID Bioinformatics Resources. *Nature Protoc. 2009;4(1):44-57*)*.*

.

www.aging-us.com 2 AGING
